# Noninvasive vagus nerve stimulation for migraine: a systematic review and meta-analysis of randomized controlled trials

**DOI:** 10.3389/fneur.2023.1190062

**Published:** 2023-05-11

**Authors:** Dong Song, Piaoyi Li, Yonggang Wang, Jin Cao

**Affiliations:** ^1^School of Life Sciences, Beijing University of Chinese Medicine, Beijing, China; ^2^Department of Neurology, The First Affiliated Hospital of Zhengzhou University, Zhengzhou, China; ^3^Department of Neurology, Beijing Tiantan Hospital, Capital Medical University, Beijing, China

**Keywords:** non-invasive vagus nerve stimulation (n-VNS), migraine, non-invasive cervical vagus nerve stimulation (n-cVNS), non-invasive auricular vagus nerve stimulation (n-aVNS), curative effect

## Abstract

**Background:**

Medication is commonly used to treat migraine. However, patients may experience adverse events or fail to respond to medication. In recent years, neuromodulation techniques have emerged as potential non-pharmacological therapy for migraine. This article focuses on a systematic review and meta-analysis of randomized controlled trials of non-invasive vagus nerve stimulation (n-VNS) for migraine to determine the efficacy, safety and tolerability of n-VNS.

**Methods:**

We searched PUBMED, EMBASE, and Cochrane Center Register of Controlled Trials databases up to July 15, 2022. Primary outcomes were monthly reduced migraine/headache days, and pain-free rates within 2 h. Secondary outcomes were  ≥ 50% responder rate, headache intensity, monthly acute medication reduction days, and adverse events.

**Results:**

Meta-analysis shows that non-invasive cervical vagus nerve stimulation (n-cVNS) significantly impacted ≥50% responder rate (OR, 1.64; 95% CI, 1.1 to 2.47; *p* = 0.02), but had no significant effect on reducing migraine days (MD, −0.46; 95% CI, −1.21 to 0.29; *p* = 0.23) and headache days (MD, −0.68; 95% CI, −1.52 to 0.16; *p* = 0.11). In contrast, low-frequency non-invasive auricular vagus nerve stimulation (n-aVNS) was found to significantly reduce the number of migraine days (MD, −1.8; 95% CI, −3.34 to −0.26; *p* = 0.02) and headache intensity (SMD, −0.7; 95% CI, −1.23 to −0.17; *p* = 0.009), but not the number of acute medication days per month (MD, −1.1; 95% CI, −3.84 to 1.64; *p* = 0.43). In addition, n-cVNS was found safe and well-tolerated in most patients.

**Conclusion:**

These findings show that n-VNS is a promising method for migraine management.

## Introduction

Migraine patients are exposed to high levels of stress and burden (1). While there’s currently no known cause or cure for migraines, pharmacologic treatments (including acute and preventive migraine medications) are commonly used in migraine intervention. However, medical treatment seems to be insufficient in migraine management due to its unsatisfactory therapeutic effects, contraindications, and side effects (2). Non-pharmacological migraine treatments have therefore drawn increased attention from researchers and the public.

Neuromodulation has been proven to be a promising non-pharmacological management for migraine (3). Compared to invasive approaches that require a surgical procedure, accumulating studies have demonstrated the efficacy and safety of non-invasive neuromodulation methods, which comprises non-invasive vagus nerve stimulation (n-VNS) (4), transcranial direct current stimulation (tDCS) (5), transcranial magnetic stimulation (TMS) (6), and external trigeminal nerve stimulation (e-TNS) (7). There are two types of n-VNS, non-invasive cervical vagus nerve stimulation (n-cVNS) and non-invasive auricular vagus nerve stimulation (n-aVNS). Specifically, n-cVNS is a promising neuromodulation approach that has been approved by the FDA for acute migraine management since 2018 (3). It modulates the vagus nerve by placing electrodes on the neck skin and delivering 1 ms pulse (5 kHz sine wave) through the handheld device GammaCore (ElectroCore, Inc. Rockaway NJ). N-aVNS can modulate the vagus nerve with electrodes placed on the auricular concha (8). It is also worth noting that frequency is a key stimulation parameter of n-aVNS. In a previous study, researchers have found that compared with 25 Hz, 1 Hz stimulation was safer and more effective in alleviating chronic migraine (9). Although there is increasing data and evidence from several randomized controlled trials using n-VNS (n-cVNS and/or n-aVNS) for the management of migraine (10), to our knowledge there have been no systematic reviews and meta-analyses that have comprehensively assessed the therapeutic efficacy, safety, and tolerability of both n-cVNS and n-aVNS for the treatment of migraine.

A recent study showed that non-invasive neuromodulation had a prominent effect on both pain relief and pain-free rates within 2 h in migraine patients (7). Another study proceeded with a systematic review and meta-analysis of six clinical studies of n-cVNS for managing migraine and cluster headache. They found that n-cVNS significantly reduced pain at 30 and 60 min after the intervention and reduced the use of acute medication, but no significant reduction in headache days was found (11). The current systematic review aims to evaluate of the clinical evidence from randomized controlled trials that investigated the therapeutic effects of n-VNS (using n-cVNS and/or n-aVNS) for the acute and preventive treatment of migraine, and to provide a reliable reference for the clinical application of n-VNS.

## Methods

We reported this systematic review and meta-analysis, adhering to the Preferred Reporting Items for Systematic Reviews and Meta-Analysis (PRISMA) guidelines (12). The review has been registered in the International Prospective Register of Systematic Reviews (PROSPERO: CRD42022352371).

Data were selected from PUBMED, EMBASE, and Cochrane Center Register of Controlled Trials databases, according to the principles of population, intervention, comparison, outcome, and setting (PICOS). Population (P): adult patients with migraine; intervention (I): n-VNS; comparison (C): control group with sham stimulation or n-VNS at other frequencies; outcomes (O): monthly reduced migraine/headache days, pain-free rates within 2 h, ≥50% responder rate, headache intensity, monthly acute medication reduction days, and adverse events; setting (S): a randomized controlled trial.

Firstly, we searched the PUBMED and Cochrane Center Register of Controlled Trials databases for eligible studies using a combination of keywords and MeSH terms of “vagus nerve stimulation, migraine disorders, and randomized controlled trials (RCT).” Meanwhile, we also searched the free words in PUBMED databases, which are almost all MeSH terms, to widen the scope of the search and include as many eligible studies as possible. Free words included “vagus nerve stimulations,” “nerve stimulation, vagus,” “nerve stimulations, vagus,” “stimulation, vagus nerve,” “stimulations, vagus nerve,” “nerve stimulation, vagal,” “nerve stimulations, vagal,” “stimulation, vagal nerve,” “stimulations, vagal nerve,” “vagal nerve stimulation,” “vagal nerve stimulations,” “migraine disorder,” “disorder, migraine,” “disorders, migraine,” “migraine,” “migraines,” “migraine headache,” “migraine headaches,” “headache, migraine,” “headaches, migraine,” “acute confusional migraine,” “acute confusional migraines,” “status migrainosus,” “hemicrania migraine,” “hemicrania migraines,” “migraine variant,” “sick headache,” “headache, sick,” “abdominal migraine,” “cervical migraine syndrome,” “migraine syndrome, cervical” and so on. The search strategies for RCT were based on the standard set by evidence-based medicine secondary schools of McMaster University. In EMBASE databases, we combined the Emtree terms of “vagus nerve stimulation, migraine disorders, and RCT,” and also searched the free words from PUBMED. Secondly, we exported the literatures retrieved to Endnote, including abstracts, reviews, and studies. Finally, we used Endnote to remove duplicate literatures as well as the reviews and literatures with only abstract according to the title and abstract. From the remaining studies, we screened out studies with the following eligibility criteria.

### Eligibility criteria

We included studies that met the following criteria based on the clinical research guidelines for the management of migraine (13–16): (1) population diagnosed with migraine but without other primary and/or secondary headaches according to international standards (ICDH or IHS), (2) n-VNS intervention, (3) randomized controlled trials, (4) double-blind period for at least 4 weeks, and (5) language in English.

We excluded studies that met the following criteria: (1) non-randomized controlled trials, (2) invasive vagus nerve stimulation, and (3) studies with unpublished findings or insufficient data.

### Data collection

Two researchers (DS and PL) screened the eligible studies independently. Any conflict was agreed upon. If an agreement could not be reached, a third party was consulted until an agreement was reached. The two researchers extracted the following information — first author, year of the research, the type, age, the number of the population investigated, the number of females in each group, adverse events, and outcomes. Primary outcomes included monthly reduced migraine/headache days, and pain-free rates within 2 h. Secondary outcomes included ≥50% responder rate, headache intensity, monthly acute medication reduction days, and adverse events. If multiple papers reported on one study, we cited one of them, but the results of the outcomes included in all papers were statistically analyzed to ensure complete inclusion of the study results. Any conflict was agreed upon. If an agreement could not be reached, we would consult a third party until an agreement was reached.

According to the clinical research guidelines for the management of migraine (13–15), a migraine day is defined as 24 h with headache lasting at least 30 min without the intake of analgesics and meeting criteria for migraine or probable migraine in the edition of the International Classification of Headache Disorders criteria, or a day in which a migraine-specific medication (e.g., ergotamine, triptan, ditan, and gepant) respond successfully to an acute headache. Further, a moderate or severe headache day is a 24-h period in which moderate or severe headache pain lasts for at least 4 h without medical treatment, or when at least moderate headache occurs and responds to acute treatment with a migraine-specific medication. The definition of the responder rate is the percentage change from baseline in the number of migraine days or the number of moderate/severe headache days at each intervention interval.

### Risk of bias

We estimated the risk of bias and assessed the reliability of the evidence in eligible studies by applying Cochrane Risk of Bias Assessment Tool (17), which measures bias across five dimensions — selection bias, performance bias, detection bias, attrition bias, and reporting bias. Selection bias includes random sequence generation and allocation concealment. Performance bias depends on the extent to which patients and personnel are aware of the allocated interventions during the study. Attrition bias hinges on the amount, nature, or handling of the incomplete outcome data. Reporting bias depends on selective outcome reporting. The measure basically provides an overall score of every dimension, classified as “low risk,” “unclear risk,” and “high risk.” If the studies mentioned that the studies were a randomly generated sequence, we estimated the selection bias as low risk. If the method of sequence generation was not mentioned, we estimated the selection bias as unclear risk. When patients were aware of the allocated interventions, we would assess performance bias as high risk. Detection bias was low risk if outcome assessors were without clear knowledge of allocated interventions. If the researchers reported why the participants withdrew and how the data were handled, attrition bias was assessed as low risk. If the reported outcomes were complete, we would estimate reporting bias as low risk.

### Data analysis

We used the open-source software Review Manager (RevMan) version 5.4 for data synthesis and plotting. Data types were divided into dichotomous and continuous variables. For data with dichotomous variables, we applied the Inverse Variance (IV) method, Odds Ratio (OR), and the Random Effects model for data analysis. When the data were continuous variables, we adopted the Inverse Variance (IV) method, the Random Effects model, Mean Difference (MD), Std. Mean Difference (SMD) and 95% confidence interval (CI) for analysis. A two-tailed *p* < 0.05 difference was deemed to be statistically significant. Heterogeneity was evaluated by the Chi-square test and was quantified with the I^2^ statistic. To control the heterogeneity in the intervention and control groups, we reported each outcome separately, along with the results for different stimulation types (e.g., high vs. sham frequency for n-cVNS, and low vs. sham frequency for n-aVNS).

## Results

### Search results

**Figure 1:**
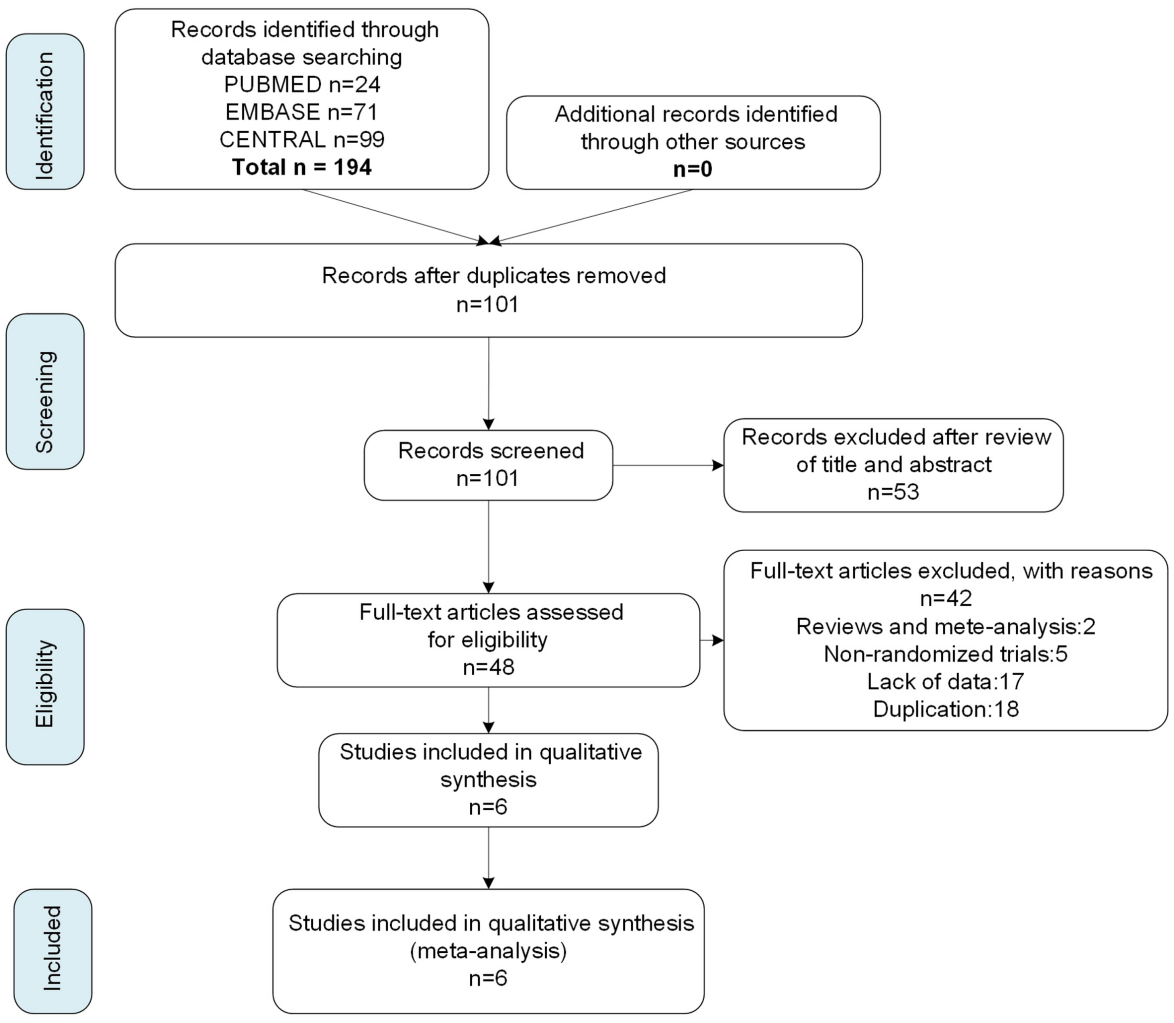
Flow diagram of study selection.

We searched 194 articles from three databases, PUBMED, EMBASE, and Cochrane Center Register of Controlled Trials, with the most recent study included on July 15, 2022. From the 194 articles, we screened six studies with published research results on the basis of eligibility criteria (Figure 1), four of which were n-cVNS studies (18–21), and the other two studied on n-aVNS (9, 22). Of the four n-cVNS studies, one was a clinical study on chronic migraine, two were clinical studies on episodic migraine, and one did not restrict the subtype (s) of migraine. As for the n-aVNS studies, one study was on chronic migraine patients and the other showed unrestricted subtypes to the migraineurs. A total of 845 patients with migraine were included in the six studies. The basic characteristics of the six eligible studies were summarized in Table 1.

**Table 1:** Characteristics of the included studies.

Author	Migraine type	Intervention	Intervention time	Groups (*n*)	Mean age	F (Intervention)/ F (Control)
Silberstein et al. (20)	Chronic migraine	n-cVNS	2 months	Intervention (*n* = 30)	39.2 years	26/27
				Sham (*n* = 29)		
Grazzi et al. (19)	Episodic migraine	n-cVNS	4 weeks	Intervention (*n* = 120)	39.2 years	95/91
				Sham (*n* = 123)		
Diener et al. (18)	Episodic migraine	n-cVNS	12 weeks	Intervention (*n* = 165)	42.4 years	142/138
				Sham (*n* = 167)		
Najib et al. (21)	Migraine	n-cVNS	12 weeks	Intervention (*n* = 56)	42.5 years	49/44
				Sham (*n* = 57)		
Straube et al. (9)	Chronic migraine	n-aVNS	12 weeks	Intervention (*n* = 17)	41.5 years	13/19
				Control group (*n* = 22)		
Zhang et al. (22)	Migraine	n-aVNS	4 weeks	Intervention (*n* = 33)	30.4 years	23/23
				Sham (*n* = 26)		

n-cVNS, non-invasive cervical vagus nerve stimulation; n-aVNS, non-invasive auricular vagus nerve stimulation; F, female.

### Risk of bias for each study

**Figure 2:**
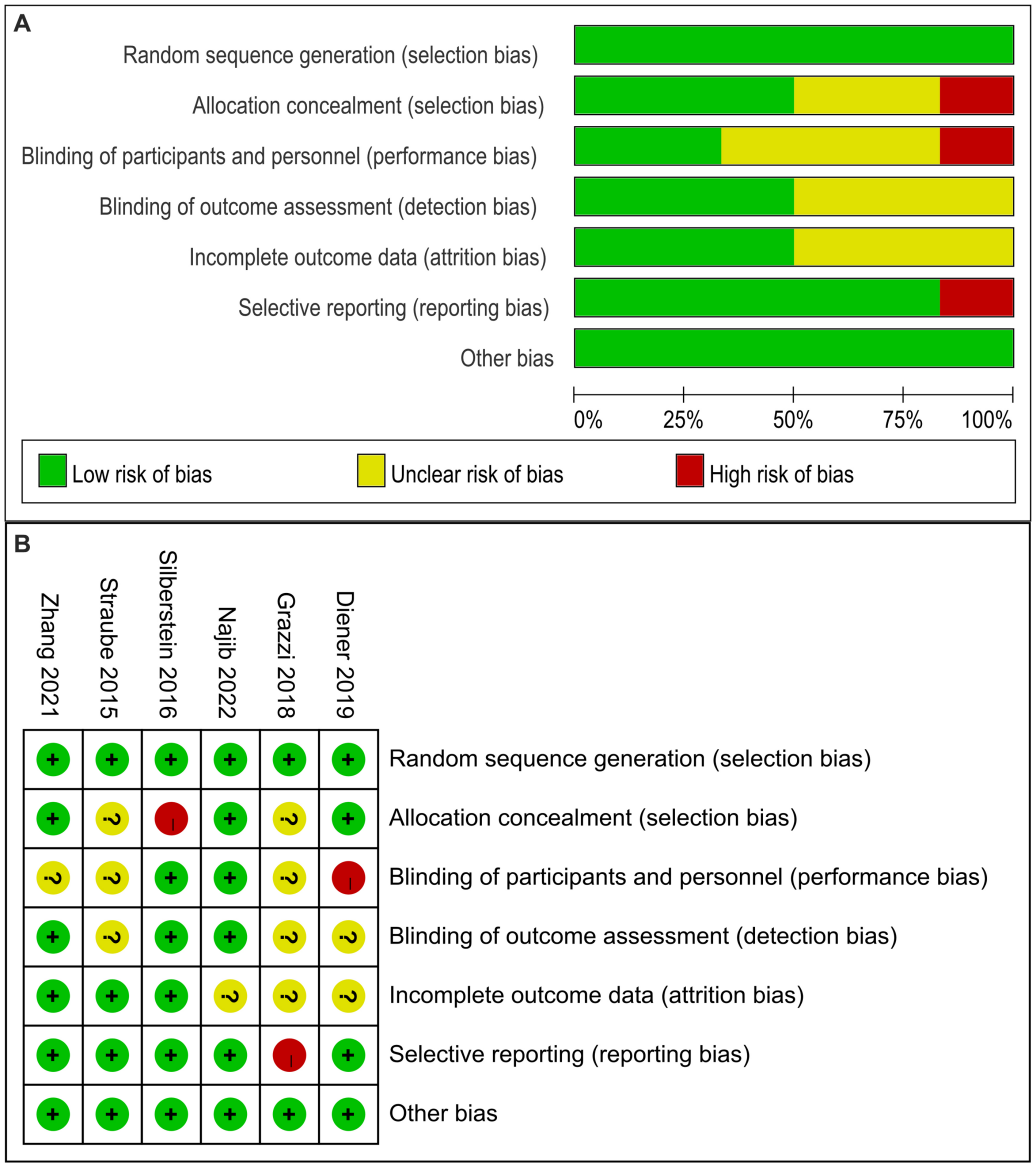
Risk of bias graph **(A)** and summary **(B)** of the 6 evaluated studies. **(A)**: Risk of bias graph: authors’ judgments about each risk of bias item presented as percentages in the review of all included studies. **(B)**: Risk of bias summary: a review of authors’ judgments about each risk of bias item for each included study.

The risk of bias results for the six eligible studies are shown in Figure 2. The results indicated that the blindness of the control group of one study might be broken (20), which could lead to a biased and misleading interpretation of the review; one study in which unblinded personnel instructed patients in the learning and use of equipment might risk unblinding the population (18); one study did not perform a complete data analysis for the end-point outcomes, but had a complete registry (19). 50% of the included clinical studies did not perform statistical analysis on whether the researchers who conducted the analysis were blinded. We found that 50% of the studies did not describe in detail the analysis of patients lost to follow-up, but the number of patients who dropped out of the study was described in the studies.

### Primary outcomes

#### Monthly reduced migraine/headache days

**Figure 3:**
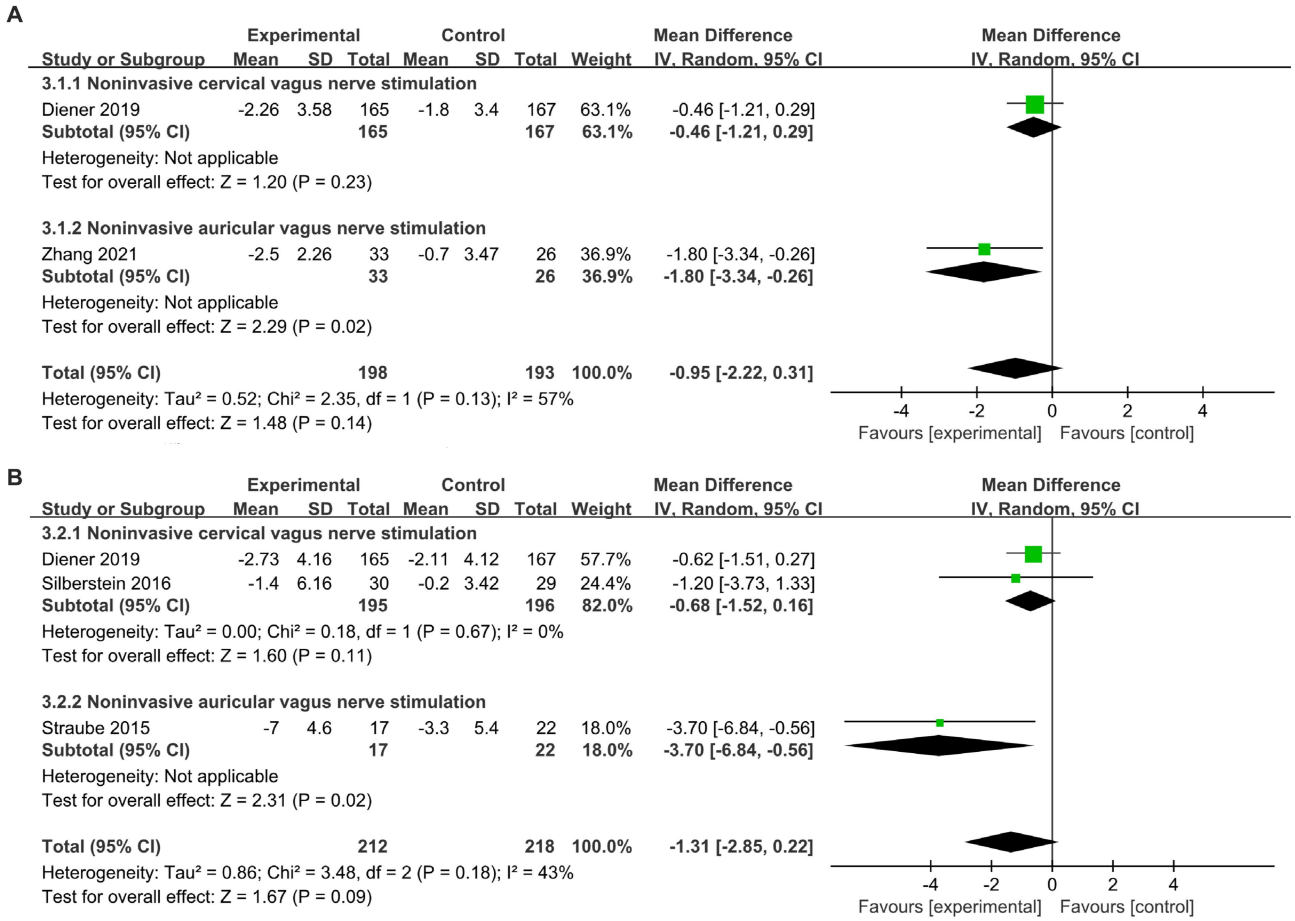
Forest plot of migraine days and headache days about n-VNS treatment for migraine. **(A)**: migraine days. **(B)**: headache days. n-VNS, non-invasive vagus nerve stimulation.

We performed subgroup analysis according to different stimulation methods. Figure 3 shows the forest plot results of the pooled meta-analysis. No significant therapeutic effect of n-VNS on monthly reduced migraine days was found (MD, −0.95; 95% CI, −2.22 to 0.31; *p* = 0.14; I^2^ = 57%). Specifically, subgroup analysis revealed that n-cVNS did not significantly reduce migraine days (MD, −0.46; 95% CI, −1.21 to 0.29; *p* = 0.23). However, compared to sham stimulation, n-aVNS significantly reduced migraine days (MD, −1.8; 95% CI, −3.34 to −0.26; *p* = 0.02) (Figure 3A).

Similarly, the forest plot of monthly reduced headache days showed that n-VNS did not reduce headache days significantly (MD, −1.31; 95% CI, −2.85 to 0.22; *p* = 0.09; I^2^ = 43%). Subgroup analysis revealed that n-cVNS could reduce the number of headache days, but the effect was statistically non-significant (MD, −0.68; 95% CI, −1.52 to 0.16; *p* = 0.11; I^2^ = 0). Only one study reported that low-frequency n-aVNS significantly reduced headache days, compared with high-frequency n-aVNS (MD, −3.7; 95% CI, −6.84 to −0.56; *p* = 0.02) (Figure 3B).

#### Pain-free rates within 2 h

**Figure 4:**
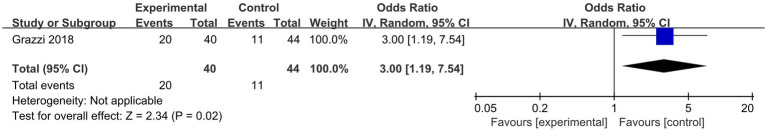
Forest plot of pain-free rates within 2 h about n-cVNS for migraine. n-cVNS, non-invasive cervical vagus nerve stimulation.

Only one study (19) has reported the primary outcome of pain-free rates within 2 h. That being said, Licia Grazzi et al.’s (19) n-cVNS study for the treatment of migraine found that if the initial pain intensity was mild, the percentage of patients with pain-free rates within 2 h was significantly higher than with sham (OR, 3.00; 95% CI, 1.19 to 7.54; *p* = 0.02) (Figure 4). The results showed that n-cVNS was effective to mild migraine.

#### Secondary outcomes: ≥50% responder rate, headache intensity, monthly acute medication reduction days

**Figure 5:**
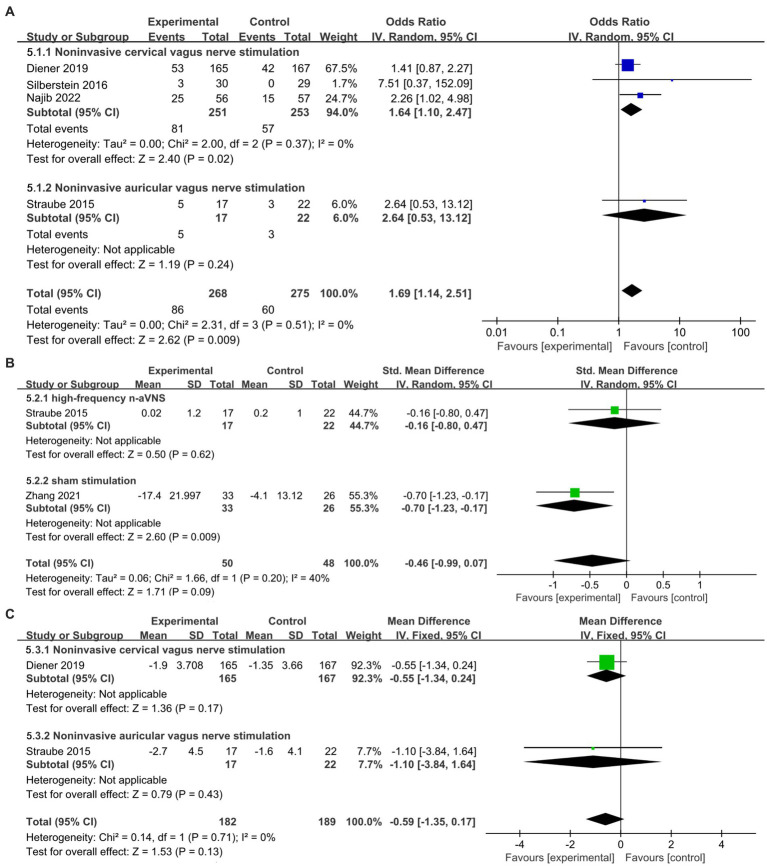
Forest plot of secondary outcomes about n-VNS for migraine. **(A)**: ≥50% responder rate about n-VNS for migraine. **(B)**: headache intensity of n-aVNS for migraine according to different control groups. **(C)**: monthly acute medication reduction days about n-VNS for migraine. n-VNS, non-invasive vagus nerve stimulation; n-aVNS, non-invasive auricular vagus nerve stimulation.

The result for ≥50% responder rate obtained by subgroup analysis based on different stimulation modalities in secondary outcomes (Figure 5A) showed that n-VNS could significantly increase ≥50% responder rate (OR, 1.69; 95% CI, 1.14 to 2.51; *p* = 0.009; I^2^ = 0%). N-cVNS for migraine obviously increased ≥50% responder rate without heterogeneity (OR, 1.64; 95% CI, 1.1 to 2.47; *p* = 0.02; I^2^ = 0); however, only one n-aVNS study reported an insignificant increase in ≥50% responder rate (OR, 2.64; 95% CI, 0.53 to 13.12; *p* = 0.24).

The forest plot of headache intensity obtained by subgroup analysis based on different control groups (Figure 5B) showed that compared with high-frequency n-aVNS, low-frequency n-aVNS did not significantly reduce headache intensity (SMD, −0.16; 95% CI, −0.8 to 0.47; *p* = 0.62). Compared to sham stimulation, low-frequency n-aVNS significantly reduced headache intensity (SMD, −0.7; 95% CI, −1.23 to −0.17; *p* = 0.009).

Figure 5C shows that n-VNS did not significantly reduce monthly acute medication use days (MD, −0.59; 95% CI, −1.35 to 0.17; *p* = 0.13; I^2^ = 0%). Subgroup analysis (Figure 5C) demonstrated that neither n-cVNS (MD, −0.55; 95% CI, −1.34 to 0.24; *p* = 0.17) nor n-aVNS (MD, −1.1; 95% CI, −3.84 to 1.64; *p* = 0.43) significantly reduced monthly acute medication use days. Only one study showed that n-aVNS reduced migraine attacks (*p* = 0.015) (22).

#### Tolerability and safety

**Figure 6:**
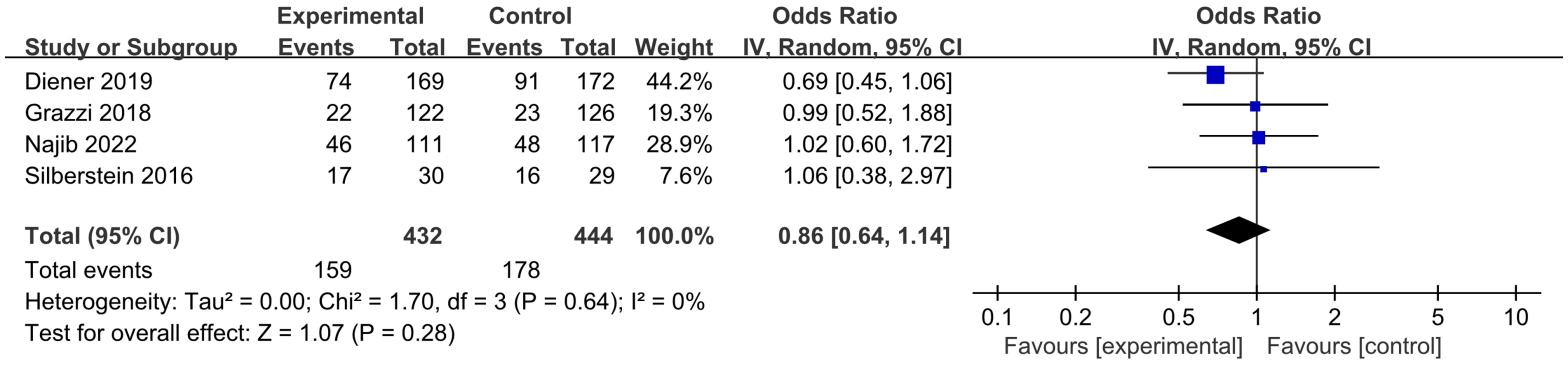
Forest plot of adverse events about n-cVNS for migraine. n-cVNS, non-invasive cervical vagus nerve stimulation.

The results (Figure 6) showed that compared to sham stimulation, n-cVNS was safe and well-tolerated in the migraineurs (OR, 0.86; 95% CI, 0.64 to 1.14; *p* = 0.28; I^2^ = 0).

## Discussion

In the current study, we applied subgroup analysis to n-cVNS and n-aVNS separately. Therefore, the study investigated not only the efficacy of each type of n-VNS, but also the efficacy of combined n-cVNS and n-aVNS based on the summary results. Also, compared with previous review (7), an update of this study is the complete inclusion of the results of n-VNS (including n-cVNS and n-aVNS). Considering vagus nerve stimulation can be used in treating both chronic and episodic migraine, this study comprehensively included both acute and preventive outcomes, thus covering research on the use of vagus nerve stimulation for treating migraine of different duration. The meta-analysis showed that n-VNS significantly reduced migraine/headache days monthly but with moderate heterogeneity. We also found that n-cVNS could significantly increase ≥50% responder rate, and low-frequency n-aVNS could significantly reduce headache intensity. In addition, no significant difference in safety and tolerability has been found between the experimental groups and the control groups.

Effective strategies for clinicians to manage migraine include acute treatment and prophylactic measures. Effective treatment is expected to provide immediate control of episodic migraine attacks in order to promote functional restoration and reduce the use of medication (23). The first line of treatment for chronic migraine is medication treatment. Although the development of anti-CGRP monoclonal antibodies has brought good news to migraine patients and studies have confirmed that anti-CGRP monoclonal antibodies play a role in preventing migraine, the cost of anti-CGRP monoclonal antibodies is high (24–30). Therefore, the safe, efficacious, and cost-effective nonpharmacological management for migraine has drawn increasing attention from clinicians, researchers, and the public.

Our findings revealed that n-VNS significantly reduced the number of migraine days and headache days. N-VNS is thus expected to be combined with medication to prevent migraine attacks. Similar to a previous meta-analysis (31) that reported safety and tolerability of n-VNS, the results of the study showed common mild side effects of n-VNS, i.e., mild or moderate pain and redness at the site of irritation and contraction of facial or neck muscles. Therefore, n-cVNS is considered safe and well-tolerated in clinical application, which shows to be a patient-friendly and safe approach for migraine patients over 65 years old (16). This conclusion is undoubtedly needed to be verified in future clinical studies (16).

Although n-VNS shows promising clinical efficacy, its neural mechanism for treating migraine remains unclear. A previous study has indicated that stimulating the vagus nerve can inhibit cortical spreading depression (CSD) (32), and regulate neurotransmitters (33, 34) and nerves (35). It makes sense that one of the potential mechanisms triggering migraine attacks is CSD (36, 37). It is a slowly propagating depolarization wave (38) that can lead to cortical hyperemia and diffuse hypoperfusion, activate the trigeminal vascular system, and result in headache (36). A previous animal study has found that both n-VNS and invasive VNS significantly inhibited the CSD in rats (an animal model of migraine with aura). The frequency of repetitive CSDs over the occipital region, which was triggered by continuous drug (KCl) application, was cut down by 40%, and the propagation speed of the spreading depression decreased by 15% (32). The pathogenesis of migraine is also related to the dysfunction of neurotransmitter regulation (39), in that researchers have found that VNS can modulate neurotransmitters in animal models of migraine (33).

A functional magnetic resonance imaging (fMRI, a noninvasive neuroimaging technique that can probe differences in brain structure and functions) study showed that 1 Hz n-aVNS could significantly modulate brain functional connectivity in key brain regions such as the motor-related thalamus subregion, anterior cingulate cortex/medial prefrontal cortex, occipital cortex-related thalamus subregion, and postcentral gyrus/precuneus. Changes in resting state functional connectivity between the occipital thalamic seed and the bilateral postcentral gyrus induced by the n-aVNS were observably negatively related to the decrease of migraine days (22). These findings suggest that vagus nerve stimulation modulates activity in different brain regions through pathways such as the nucleus solitarius and locus coeruleus, thereby regulating migraine-related symptoms.

In addition, the brainstem trigeminal nervous system directly or indirectly associated with the vagus nervous system is involved in migraine, and VNS may modulate pain by inhibiting trigeminal nervous system discharge (35). In episodic migraine model rats, previous studies have found that n-VNS and sumatriptan inhibited trigeminal nerve activation to a similar extent, increasing the inhibitory effect of descending pain pathways by increasing GABA and serotonergic nerve signaling (33). For excitatory neurotransmitters in the trigeminal nervous system, vagus nerve stimulation was found to reduce the level of glutamate in the trigeminal nerve and reduce the rats’ periorbital sensitivity in migraine model rats (34). N-VNS may affect trigeminal nerve activity by modulating excitatory and inhibitory neurotransmitters, but may also be associated with peripheral sensitization-related proteins, such as P-ERK (40) in the trigeminal nerve. These underlying mechanisms provide theoretical support for the n-VNS treatment of migraine.

Although previous studies have included various types of nerve stimulation for migraine (7) or various types of nerve stimulation for migraine and cluster headache (41), the current systematic review focuses on the efficacy and safety of vagus nerve stimulation for migraine and is more precise in scope (albeit only with six literatures being included in the review). Six clinical studies eligible for the review targeted at different endpoint outcomes. For example, three clinical studies reported ≥50% responder rate, and two studies reported headache intensity. The heterogeneity of the endpoint outcomes limited the stability of the review and the reference for future clinical applications. Therefore, we recommend that in future clinical studies of migraine, primary endpoints should include the change of migraine days, moderate to severe headache days, responder rate, and pain-free rates within 2 h, with secondary endpoints included depending on respective objectives.

## Limitations

A total of six clinical studies were included in the current study, and the sample size was relatively small to conduct a sensitivity analysis. Therefore, we individually removed each study that included in each outcome and observed the specific impact results and sensitivity of each study, without conducting statistical analysis by other methods. In addition, there are few studies using n-aVNS, one of which was not sham-controlled. More patients should be included in future double-blind clinical studies in order to assess the efficacy and security of n-aVNS in treating migraine more reliably.

## Conclusion

The current study found that n-VNS can significantly reduce migraine or headache days, n-cVNS for migraine markedly increased ≥50% responder rate, and low-frequency n-aVNS could significantly reduce headache intensity. The findings support that the potential of n-VNS to reduce disease burden and improve quality of life in migraineurs.

## Author contributions

JC and YW conceived and designed this study. DS and PL carried out the searches and performed relevant analyses. DS, YW, and JC drafted and revised the manuscript. DS, PL, YW, and JC read and approved the final manuscript. All authors contributed to the article and approved the submitted version.

## Funding

The study was supported by the StartUp Research Sponsorship Program by Beijing University of Chinese Medicine (No. 90011451310036).

## Conflict of interest

The authors declare that the research was conducted in the absence of any commercial or financial relationships that could be construed as a potential conflict of interest.

## Publisher’s note

All claims expressed in this article are solely those of the authors and do not necessarily represent those of their affiliated organizations, or those of the publisher, the editors and the reviewers. Any product that may be evaluated in this article, or claim that may be made by its manufacturer, is not guaranteed or endorsed by the publisher.

## Abbreviations

TMS: transcranial magnetic stimulation
n-VNS: non-invasive vagus nerve stimulation
tDCS: transcranial direct current stimulation
e-TNS: external trigeminal nerve stimulation
n-cVNS: non-invasive cervical vagus nerve stimulation
n-aVNS: non-invasive auricular vagus nerve stimulation
PRISMA: Systematic Reviews and Meta-Analysis
IV: Inverse Variance
MD: mean difference
CI: confidence interval
OR: odds ratio
SMD: Std. Mean Difference
CSD: cortical spreading depression
